# Goosegrass Detection in Strawberry and Tomato Using a Convolutional Neural Network

**DOI:** 10.1038/s41598-020-66505-9

**Published:** 2020-06-12

**Authors:** Shaun M. Sharpe, Arnold W. Schumann, Nathan S. Boyd

**Affiliations:** 1 Gulf Coast Research and Education Center, University of Florida, Wimauma, FL USA; 2 Citrus Research and Education Center, University of Florida, Lake Alfred, FL USA

**Keywords:** Ecological modelling, Plant sciences

## Abstract

Goosegrass is a problematic weed species in Florida vegetable plasticulture production. To reduce costs associated with goosegrass control, a post-emergence precision applicator is under development for use atop the planting beds. To facilitate *in situ* goosegrass detection and spraying, tiny- You Only Look Once 3 (YOLOv3-tiny) was evaluated as a potential detector. Two annotation techniques were evaluated: (1) annotation of the entire plant (EP) and (2) annotation of partial sections of the leaf blade (LB). For goosegrass detection in strawberry, the *F-score* was 0.75 and 0.85 for the EP and LB derived networks, respectively. For goosegrass detection in tomato, the *F-score* was 0.56 and 0.65 for the EP and LB derived networks, respectively. The LB derived networks increased *recall* at the cost of *precision*, compared to the EP derived networks. The LB annotation method demonstrated superior results within the context of production and precision spraying, ensuring more targets were sprayed with some over-spraying on false targets. The developed network provides online, real-time, and *in situ* detection capability for weed management field applications such as precision spraying and autonomous scouts.

## Introduction

Goosegrass [*Eleusine indica* (L.) Gaertn.] is an invasive and problematic weed with nearly worldwide distribution including North and South America, Africa, Europe, Australia, and Southeast Asia^1^. Goosegrass infests many agroecosystems including turfgrass^2,3^, rice^4^, and fruiting vegetable crops^5^. In Florida, goosegrass is a problematic weed in many major horticultural crops including strawberry [(*Fragaria* × *ananassa* (Weston) Duchesne ex Rozier (pro sp.) [*chiloenis* × *virginiana*]], bell pepper (*Capsicum annuum* L.), tomato (*Solanum lycopersicum* L.), and cucurbit (Cucurbitaceae) production. While goosegrass interference has not been extensively studied in horticultural crops, it has shown to interfere with cotton (*Gossypium hirsutum* L.) yield in the field^6^ and greenhouse-grown corn (*Zea mays* L.)^7^.

In Florida, many broadleaf horticultural crops are produced using a plasticulture system. This system included raised beds covered in plastic mulch with drip irrigation installed to provide nutrients and moisture. Weeds within this system primarily occur within the planting holes or between the rows, except for purple nutsedge (*Cyperus rotundus* L.) and yellow nutsedge (*Cyperus esculentus* L.) which penetrate and emerge through the plastic mulch.

Within vegetable horticulture, the prevalent post-emergence weed management options for goosegrass control include hand weeding and herbicides. For pre-plant burn down and within row middles, broad-spectrum herbicides such as paraquat and glyphosate are widely employed. Consequently, both goosegrass and American black nightshade (*Solanum americanum* Mill.) have developed paraquat resistance^8,9^ and ragweed parthenium (*Parthenium hysterophorous* L.) developed glyphosate resistance^10^. For weed control atop the bed during the cropping cycle, WSSA Group 1 herbicides are the most common post-emergence chemical control option. Group 1 herbicides are becoming increasingly utilized within herbicide mixtures for grass control in row middles depending on weed pressures and resistance issues faced.

Implementing precision technology into spraying equipment is a viable option to reduce production costs associated with weed management. Goosegrass and other grass species are excellent targets for precision technology to apply Group 1 herbicides to a variety of broadleaf crops. A prototype precision sprayer was developed to simultaneously detect and spray weeds in plasticulture production within Florida. Briefly, the system was a modified plot sprayer with a digital camera sensor, a controller linked with artificial intelligence for detection, and nozzles controlled by solenoids. The desirable detector for this system is a convolutional neural network.

Machine vision-based weed detection is typically conducted using either multispectral/hyperspectral or RGB imagery, the latter being more desirable for economic costs and practical adoption for producers^11^. Recent technological advances in graphical processing units permit training and employing deep learning convolutional neural networks as detectors^12^. Deep convolutional neural network frameworks have been reviewed elsewhere^12,13^. Briefly, neural networks take inspiration from the visual cortex, containing layers for feature extraction, convolution, pooling, activation functions, and class labeling^14^. The system relies on pattern recognition via filters within the convolutional layers for detection and classification^15^. Convolutional neural networks for weed detection have been employed in several crops including turfgrass^16,17^, wheat^18^, and strawberry^19^. For horticultural plasticulture row middles, a convolutional neural network has been developed to detect grasses among broadleaves and sedges^20^. Within broader agriculture, deep neural network applications include strawberry yield prediction^21^, sweet pepper (*Capsicum annum* L.), and cantaloupe (*Cucumis melo* var. *cantalupo* Ser.) fruit detection^22^, and detection of tomato pests and diseases^23^.

With the widespread registration of Group 1 herbicides in broadleaf crops and the widespread distribution of goosegrass, the successful development of a detection network would have far-reaching implications for conventional horticulture. Development of a multi-crop, within-crop grass detection network has challenges including training image availability, ease of image collection due to the patchy nature of weeds, and the diverse background of several crops as the negative space. Additionally, the goosegrass within-crop growth habit, as well as the general habit of grassy weeds causes issues for bounding box-based network training. Goosegrass has a tufted plant habit with stems that are erect to spreading and up to 8.5 m tall, and leaves which are 5 cm to 35 cm long and 3 mm to 5 mm wide^24^. For strawberry production, goosegrass leaves have been observed to either penetrate through the crop canopy, growing prostrate along with the plastic, or grow in planting holes where strawberry plants have died. For tomato production, goosegrass plants typically grow at the base of the tomato plants, which are vertically staked for fresh-market production. The study objectives were to (1) develop a network with utilities in multiple broadleaf crops, starting with strawberry and tomato plasticulture, (2) evaluate the use of small label annotation boxes along the leaf-blade length for goosegrass detection compared to boxes encompassing the entire plant habit, and (3) evaluate a piecemeal oversampling technique.

## Results

For strawberry production, the entire plant annotation method (EP) (*precision* = 0.93; *recall* = 0.88; *F-score* = 0.90; *accuracy* = 0.82) resulted in an overall increased YOLOv3-tiny training fit compared to the leaf-blade annotation method (LB) (*precision* = 0.39; *recall* = 0.55; *F-score* = 0.46; *accuracy* = 0.30) (Table 1). Convergence time, in iterations, declined rapidly for EP compared to LB (data not shown). This was expected since EP resulted in fewer bounding boxes and provided larger bounding boxes with a static location. Labeling of goosegrass leaf blades with narrow squares resulted in “ground truth fluidity” with resultant increased training time and reduced fit.

**Table 1:** Training accuracy for network assessment of two convolutional neural networks trained to detect goosegrass developed in Balm, FL, USA in 2018^a^.

Measure	Network accuracy	
	Leaf-blade annotation	Entire plant annotation
True positives	1495	77
False positives	2367	6
False negatives	1202	11
average IoU (%)	25.10	76.12

^a^Threshold for detection was 0.25 or 25% intersection of union between the predicted and ground truth bounding box.

**Figure 1:**
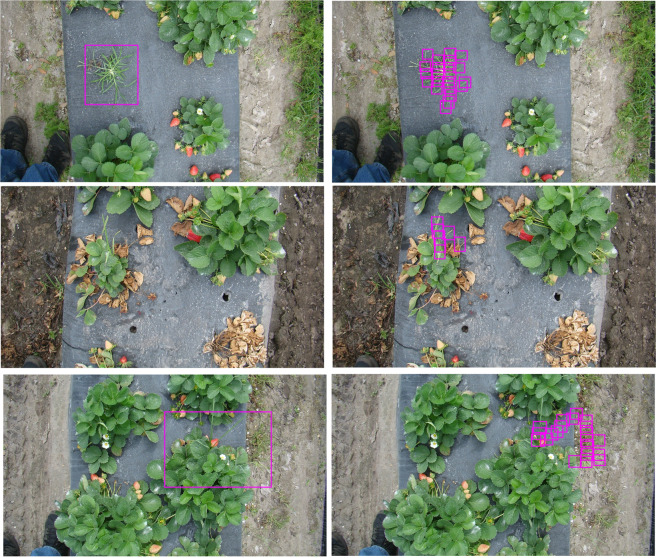
Examples of YOLOv3-tiny network detection of goosegrass (*Eleusine indica*) growing in competition with strawberry (*Fragaria* × *ananassa*) using either entire plant (left) or leaf blade (right) annotation techniques in Central FL, USA in 2018.

While the EP network appeared more successful in training, the network provided inadequate testing results. For goosegrass detection within strawberries, the LB (*precision* = 0.87; *recall* = 0.84; *F-score* = 0.85; *accuracy* = 0.74) outperformed the EP (*precision* = 0.93; *recall* = 0.62; *F-score* = 0.75; *accuracy* = 0.60) in terms of overall *F-score* and *accuracy* (Table 2). The EP method demonstrated high precision but tended to miss targets (Fig. 1). There was no impact of the annotation method on iteration time (Table 3). Compared to the EP, the LB network increased *recall* substantially at the expense of *precision* but resulted in the highest *F-score*.

**Table 2:** Pooled relevant binary classification categories and neural network accuracy measures for goosegrass (*Eleusine indica*) detection in tomato (*Solanum lycopersicum*) and strawberry (*Fragaria* × *ananassa*) using two annotation methods on digital photography acquired in Central Florida, USA, in 2018 and 2019^a^.

Measure	Network accuracy			
	Strawberry		Tomato	
	EP^b^	LB^c^	EP	LB
True positives	43	58	10	17
False positives	3	9	3	12
False negatives	26	11	13	6

^a^The neural network was the tiny version of the state-of-the-art object detection convolutional neural network You Only Look Once Version 3 (Redmon and Farhadi 2018).

^b^EP = Entire plant annotation method. This refers to using a single, large square box to identify goosegrass within digital images.

^c^LB = Leaf-blade annotation method. This refers to using multiple, small square boxes placed along leaf blades and inflorescence to identify goosegrass within digital images.

**Table 3:** Impact of annotation style on testing iteration time for goosegrass (*Eleusine indica*) detection in strawberry (*Fragaria* × *ananassa*) and tomato (*Solanum lycopersicum*) production using a convolutional neural network developed at Balm, FL, USA in 2018^a^.

Annotation style	Crop	Mean iteration time (s image^−1^)	Sample size	Standard error	Confidence interval^b^
LB^b^	Strawberry	0.008067	62	0.000263	0.007542, 0.008593
EP^c^	Strawberry	0.008125	62	0.000250	0.007625, 0.008624
LB	Tomato	0.011399	47	0.003664	0.004027, 0.018771
EP	Tomato	0.007601	47	0.000328	0.006941, 0.008261

^a^The neural network was the tiny version of the state-of-the-art object detection convolutional neural network You Only Look Once Version 3 (Redmon and Farhadi 2018).

^b^LB = Leaf-blade annotation method. This refers to using multiple, small square boxes placed along leaf blades and inflorescence to identify goosegrass within digital images.

^c^Entire plant refers to the annotation method where a single, large square box to was used to identify goosegrass within digital images.

**Figure 2:**
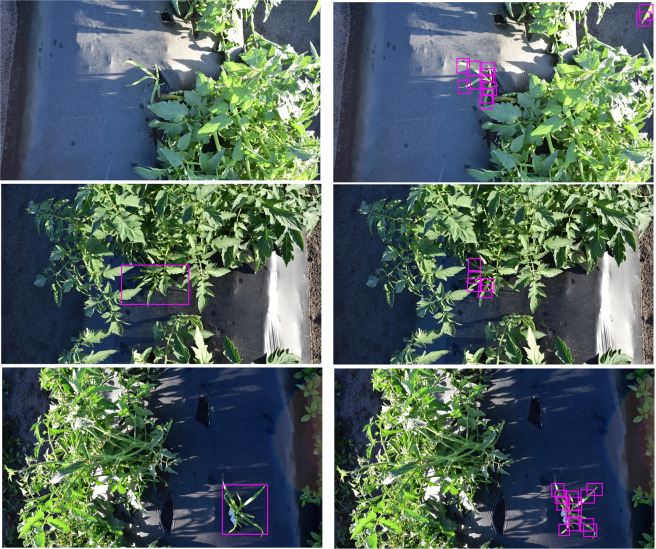
Examples of YOLOv3-tiny network detection of goosegrass (*Eleusine indica*) growing in competition with tomatoes (*Solanum lycopersicum*) using either entire plant (left) or leaf blade (right) annotation techniques in Balm, FL, USA in 2019.

For goosegrass detection in tomato, the EP (*precision* = 0.77; *recall* = 0.43; *F-score* = 0.56; *accuracy* = 0.38) had higher *precision* but struggled at detecting plants (Table 2, Fig. 2). Comparatively, the LB (*precision* = 0.59; *recall* = 0.74; *F-score* = 0.65; *accuracy* = 0.49) had reduced *precision* but had an increased *recall*. The LB derived network resulted in the highest overall *F-score* and *accuracy* for goosegrass detection in tomato.

## Discussion

Detection in strawberry production demonstrated suitable identification of goosegrass. For images taken within tomato production, success was limited (Table 2). This was most likely a consequence of available goosegrass training images within strawberry production but not for tomatoes. While attempts were made to match image acquisition angles and growth stages for both goosegrass and tomato growing in isolation, not having additional training images of the desired target and background together was likely detrimental. This could be due to the degree of actual overlap between the crop and weed in competition, altered growth habit by the weed in competition, or natural variability in the tomato growth habit inducing a stoichiometric effect that requires additional training images to overcome.

For detection in both tomato and strawberry, the LB outperformed the EP in terms of *recall*, *F-score*, and *accuracy*. The EP networks had consistently higher *precision* but lower *recall*. This was likely a consequence of selecting the entire plant habit, increasing the variability between targets, and reducing the number of potential targets for training. Such *precision* and *recall* neural network trade-offs have been noted elsewhere, including polyp detection^25^. For precision spraying, the EP network would miss many plants but would typically spray goosegrass only. Comparatively, the LB network would spray goosegrass more regularly with some degree of over-spraying upon undesirable targets. For weed detection in occluded winter wheat using a convolutional neural network based on DetectNet achieved 87% *precision* and 46% *recall*^18^. Comparatively, using an object detection convolutional neural network based on You Only Look Once to detect weeds in winter wheat images resulted in 76% *precision* and 60% *recall*^26^. Detection of Carolina geranium in strawberry using DetectNet and leaf-level annotation resulted in 99% *precision* and 78% *recall*^19^. Current results for goosegrass detection in strawberry obtained a relatively similar overall *accuracy* compared to similar studies using convolutional neural networks alone, but detection in tomatoes may require further sampling.

Results indicate the potential for a unified network for use across multiple crops. Additional options for precision spraying multi-crop networks include Group 2 herbicides in vegetable plasticulture, group 4 within cereals, and groups 9 and 10 within associated genetically modified crops. While the piecewise image methodology results for tomatoes were limited, network desensitization for additional crops does provide some benefit. Existing networks for goosegrass can be expanded to additional crops and the number of necessary training images should be reduced.

Several kinds of grass infest vegetable fields. Since the network did not classify tropical signalgrass [*Urochloa distachya* (L.) T.Q. Nguyen] as goosegrass (data not shown), additional classes are likely necessary or grouping multiple grass species into a single category^20^. A similar network (YOLOv3) was trained to detect broadleaf species that were not previously part of the training dataset^20^, so this option may be feasible but requires further study. If such is desirable, care should be taken to avoid class imbalances, which negatively impact network performance^27^,^28^.

Network performance enhancement within limited datasets may be improved using convolutional neural networks with traditional machine learning systems (support vector machines), as demonstrated with black nightshade (*Solanum lycopersicum* L.) and velvetleaf (*Abutilon theophrasti* Medik) in tomato and cotton (*Gossypium hirsutum* L.)^29^. The integration of segmentation techniques with neural networks has previously been successful and may help improve *precision* and *recall*^30,31^. For example, a weed detection system using blob segmentation and a convolutional neural network achieved 89% weed detection accuracy^32^. For some weed management scenarios, a resource such as CropDeep and DeepWeeds could be used for pre-training or supplementing datasets^33,34^. Using k-means pre-training may improve detection, which improved detection of an image classification convolutional neural network resulted from 2%, up to 93% *accuracy*^35^.

The developed networks demonstrated detection across two broadleaf vegetable crops within vegetable plasticulture production. The LB annotation technique provided superior results for goosegrass detection in strawberry production (*F-score* = 0.85) compared to the EP annotation technique (*F-score* = 0.75). Supplementing the model with a majority of isolated tomato and goosegrass images produced moderate results. The LB annotation technique provided better detection (*F-score* = 0.65) compared to the EP technique (*F-score* = 0.56). Results demonstrate that the use of the piecemeal technique alone does not provide adequate detection for field-level evaluation but may represent a suitable oversampling strategy to supplement datasets. The developed network provides an online, real-time, and *in situ* detection capability for weed management field applications such as precision spraying and autonomous scouts.

## Methods

Images were acquired with either a Sony (DSC-HX1, Sony Cyber-shot Digital Still Camera, Sony, Minato, Tonky, Japan) or Nikon digital camera (D3400 with AF-P DX NIKKOR 18-55 mm f3.5-5.6 G VR lenses, Nikon Inc., Melville NY). Training images were taken at the Gulf Coast Research and Education Center (GCREC) in Balm, FL (27.76°N, 82.22°W) and the Strawberry Growers Association (SGA) field site in Dover, FL (28.02°N, 82.23°W). Images were acquired from the perspective of the modified plot sprayer camera (T-30G-6, Bellspray, Inc., Opelousas, LA).

Training data (Training 1, Table 4) were acquired during the strawberry growing season at GCREC and SGA. Images were taken in tandem with a previous study^19^. Strawberry plants were transplanted on October 10, 2017, and October 16, 2017, at the GCREC and SGA, respectively. Several datasets were acquired due to limited goosegrass emergence at GCREC, so a piecemeal solution was undertaken. Training images of tomatoes and goosegrass were acquired separately within a plasticulture setting. A training dataset was developed for goosegrass competing with tomatoes (Training 2, Table 4). Goosegrass was grown in isolation (Training 3, Table 4), with seedlings transplanted on March 12, 2018, and May 15, 2018. Images of only tomato plants were collected for network desensitization (Training 4, Table 4). After preliminary testing, additional images were collected for network desensitization for purple nutsedge (Desensitization, Table 4), which was at the 3-leaf stage, before blooming.

**Table 4:** Training, desensitization, and testing dataset specifications for developing a convolutional neural network to detect goosegrass (*Eleusine indica*) in Florida strawberry (*Fragaria* × *ananassa*) and tomato (*Solanum lycopersicum*) production.

Dataset Type	Species	Image No.	Date	Location
Training 1	Strawberry, goosegrass	954	11 Dec 2017 to 23 Feb 2018	GCREC, SGA
Training 2	Tomato, goosegrass	28	1 May 2018 to 8 May 2018	GCREC
Training 3	Goosegrass	516	18 Mar 2018 to 29 May 2018	GCREC
Training 4	Tomato	94	4 Oct 2018	GCREC
Desensitization	Purple nutsedge	138	11 Mar 2019	GCREC
Testing 1	Strawberry, goosegrass	43	23 Feb 2018	Commercial farms
Testing 2	Strawberry, goosegrass	7	17 Dec 2018	GCREC
Testing 3	Tomato, goosegrass	60	10 Apr 2019	GCREC
Testing 4	Tomato, goosegrass	27	4 Oct 2018	GCREC
Testing 5	Goosegrass	12	14 Mar 2019	GCREC

Abbreviations: GCREC = Gulf Coast Research and Education Center at Balm, FL; SGA = Strawberry Growers Association field site in Dover, FL.

Five datasets were acquired for network testing to meet each crop objective and provide sufficient samples. Images were collected at two commercial strawberry farms (27.93°N, 82.10°W, and 27.98°N, 82.10°W) (Testing 1, Table 4) and supplemented with images from GCREC (Testing 3, Table 4). Images were collected approximately 134 and 136 days after strawberry transplanting from commercial farms and 60 days after transplanting at GCREC. For testing images in tomato production (Testing 3, Table 4), goosegrass seedlings (approximately 5-leaf stage) were transplanted into planting holes containing tomato plants transplanted on March 4, 2019. The tomato data was supplemented with additional tomato images (Testing 4, Table 4) to evaluate the network’s ability to discriminate goosegrass from another grass species. A fifth dataset included goosegrass growing in isolation (Testing 5, Table 4).

The image resolution of the Nikon digital camera was 4000 × 3000 pixels. Nikon images were resized to 1280 × 853 pixels and cropped to 1280 × 720 pixels (720p) using IrfanView (Version 4.50, Irfan Skiijan, Jajce, Bosnia). The Sony digital camera image resolution was 1920 × 1080 pixels and images were resized to 720p. Training images were annotated using custom software compiled with Lazarus (https://www.lazarus-ide.org/) in two ways. The EP annotation method used a single bounding box to encompass the entire plant habit. The LB annotation method used smaller bounding boxes along the leaf blade to reduce the overall variability of the target. This approach had been utilized previously to improve detection by focusing annotation to individual Carolina geranium (*Geranium carolinianum* L.) leaves^19^. Due to the leaf shape and potential goosegrass leaf angles, square bounding boxes were not an ideal solution to minimize background noise by annotating entire leaves. Instead, multiple small square bounding boxes, approximately the width of the leaves, were used to label goosegrass along the length of the leaves. Examples of each method are matched by corresponding bounding box output found in Figs. 1 and 2. Bounding box annotation was the preferable technique compared to pixel-wise annotation due to increased accuracy and reduced time consideration^22^.

The convolutional neural network utilized was tiny- You Only Look Once Version 3 (YOLOv3-tiny)^36^. YOLOv3-tiny was selected for the implementation into a developed prototype precision sprayer for *in situ* spraying of grasses in horticultural crops including strawberry and tomato plasticulture. The sprayer has a 50 cm distance between the camera and the solenoid-controlled nozzles. As such, image processing speed was considered a priority. The state-of-the-art object detection neural network for iteration speed and capacity for implementation into the controller was selected.

YOLOv3-tiny feature extraction is achieved with the convolutional-based Darknet-19^36,37^. Darknet-19 was derived for YOLOv2, using 3 × 3 filters within its 19 convolutional layers and 1 × 1 filters within its 5 max-pooling layers^38^. Localization is achieved by dividing the image into a grid, predicting multiple bounding boxes within each, and using regression to resolve spatially separated predictions^39^. Bounding box classification permits multiple classification categories and multi-labeling of predictions^37,39^, which is particularly useful for mixed weed communities.

YOLOv3-tiny was trained and tested using the Darknet infrastructure^40^ and pre-trained with the COCO dataset^41^. YOLOv3-tiny contained augmentation parameters to reduce the opportunity for overtraining on irrelevant features through altering input images. These parameters included color alteration (exposure, hue, and saturation), flipping, cropping, and resizing. Network training continued until either the average loss error stopped decreasing or the validation accuracy (recall or precision) stopped increasing. For training, 10% of the available images were randomly selected as the validation dataset used during training.

To assess network effectiveness, classification output was pooled and categorized by binary classification for networks derived from both annotation methods. These categories included true positives (*tp*), false positives (*fp*), and false negatives (*fn*). A *tp* was when the network correctly identified the target. An *fp* was when the network falsely predicted the target. An *fn* was when the network failed to predict the true target. *Precision*, *recall*, *F-score*, and *accuracy* were used to evaluate the network effectiveness to predict targets^12^. *Precision* measures the effectiveness of the network in properly identifying its target and was calculated as^39,40^:

1 $$Precision=\,\frac{tp}{tp+fp}$$

*Recall* evaluates the effectiveness of the network in target detection and was calculated as^42,43^:

2 $$Recall=\,\frac{tp}{tp+fn}$$

The *F-score* is the *precision* and *recall* harmonic mean and gives an overall performance measure with considerations to both *fp* and *fn*, and is calculated as^42^:

3 $$F-score=\,\frac{2\,\ast \,Precision\,\ast \,Recall}{Precision+Recall}$$

For comparison purposes, the testing network accuracy was calculated as:

4 $$Accuracy=\,\frac{tp}{tp+fp+fn}$$

To validate the network training fit, the “map” command was specified. This method used an intersection of union (IoU) with a threshold of 0.25 to evaluate predicted estimates compared to ground-truth annotation. This measure was included to evaluate the effectiveness of the annotation method on overall training. For network detection accuracy assessment of testing datasets, a separate approach was taken for precision sprayer considerations. For both annotation methods, should any of the plant falls within the predicted bounding box, it was considered a hit (IoU > 0). Additional predicted bounding boxes on the same plant were ignored. This method prioritized the detection of some part of the goosegrass plant and is reliant on the ability of the controller software to compensate and increase the area sprayed if necessary.
